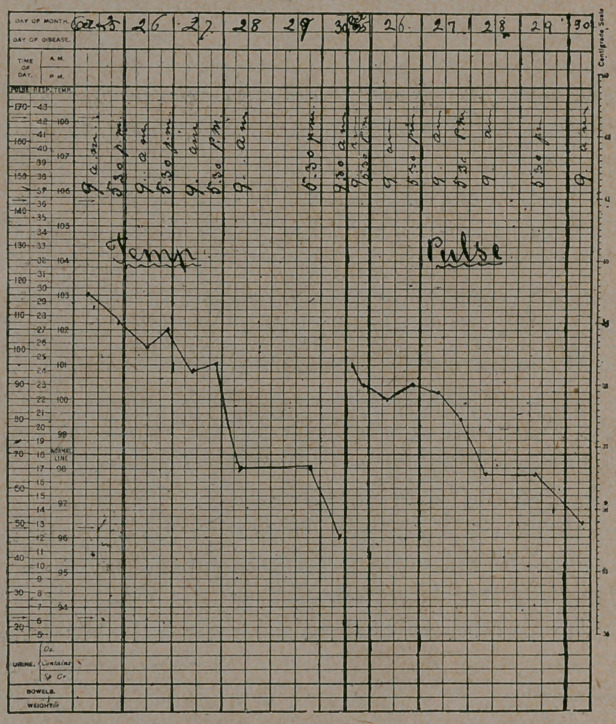# The Differential Diagnosis between Dengue and Yellow Fever with Some Account of the Epidemic of 1897 in Texas*Read at meeting of Texas State Medical Association at Houston, Texas, April 1898. Reprint from Transactions, 1897-8.

**Published:** 1898-11

**Authors:** H. A. West

**Affiliations:** Galveston, Texas; Ex-Professor Practice, Texas Medical College


### TEXAS MEDICAL JOURNAL.

ESTABLISHED JULY, 1885.

PUBLISHED MONTHLY.—SUBSCRIPTION $1.00 A YEAR.

VOL. XIV. AUSTIN, NOVEMBER, 1898. No. 5.

### Original Contributions.

### The Differential Diagnosis Between Dengue and Yellow Fever With Some Account of the Epidemic of 1897 in Texas.*

H. A. WEST, M. D., GALVESTON, TEXAS.
Ex-Professor Practice, Texas Medical College.

The diagnosis of disease is universally and justly regared as the foundation stone upon which rests the entire superstructure of practical medicine. The question concerns the health, life or death of the individual affected, his family and friends, the public in so far as he may be a useful and influential member of society, and the physician as affording a basis for his prognosis and treatment. But there are occasions when far more momentous interests are involved in the correct diagnosis of prevailing diseases: a complete paralysis of commerce, the wheels of industry arrested, enforced idleness with consequent poverty and suffering of thousands, enormous depreciation in property values of every kind, universal fear and panic, and the possibility of wide-spread death and desolation. To the physician himself an error in diagnosis ordinarily involves results which are comparatively inconsequential; on the other hand, there are times when such an error means public denunciation and disapprobation. A correct diagnosis, even when opposed to commercial interests, may involve him in losses of reputation and business which may last for a lifetime.

*Read at meeting of Texas State Medical Association at Houston, Texas, April 1898. Reprint from Transactions, 1897-8.

This was the state of affairs in Texas during the summer and autumn of 1897. So long as yellow fever was supposed to prevail east of the Mississippi only, and that dengue was the epidemic disease in Texas, peace reigned; but when the trouble came to our doors by the announcement of yellow fever in Galveston and Houston, there came the contest over the question of diagnosis, with visits of local experts from city to city, the gradual passing of the epidemic with no record of deaths from yellow fever, and the popular conviction of a stupendous error committed by those who believed in the presence of that disease. The majority, both of physicians and laymen in the State are firm in the conviction that dengue was the disease which was prevalent in Texas during the past summer; a minority believe that yellow fever also prevailed, but that owing to its mild form, indisposition to spread, exceptionally small mortality and resemblance to dengue was usually unrecognized. Confusion has arisen in the minds of many. They ask themselves, "Are the land-marks all swept away? Have the authorities led us astray? Has a new disease, *anomalous dengue*, made its appearance? Is there no such thing as dengue? Is the so-called dengue a mild form of yellow fever? Does the latter arise de novo?"

The important issues dependent upon a knowledge and recognition of the truth upon these points render it not only pertinent but imperative upon the part of those who are familiar with the facts to study them in concert. Now that the obscuring mists due to commercialism, prejudice and passion, have for the most part passed away, we approach the subject from a scientific standpoint. It is with this end in view and in no spirit of dogmatism or self-assertion that I introduce the subject for consideration. The following hypotheses have been assumed in relation to the recent epidemic:

1. The disease was dengue only. There was no yellow fever in Galveston, Houston, or the State of Texas in 1897.

2. There were anomalous cases of dengue, presenting all the symptoms of yellow fever, but proven not to be that disease, by the indisposition to spread from numerous foci and the low mortality rate.

3. During the progress of an intense epidemic of dengue throughout the State, in Galveston, Houston, and possibly other places, yellow fever made its appearance, and in consequence of its mild form and resemblance to the prevalent disease was generally unrecognized.

4. An imputed hypothesis that the epidemic of 1897 in Texas was yellow fever only.

5. A few cases, terminating fatally, and others attended by marked jaundice and albuminuria were denominated acute infectious jaundice (Weil's disease).

In order to obtain definite information upon the subject, the following circular of inquiry was distributed to a limited extent. I regret very much that replies could not have been obtained from every infected place in the State, so as to have made the report an exhaustive one. The subjoined tabular statement will, I trust, afford sufficient data upon which to base some definite conclusions. Following is the circular of inquiry:

"It is my intention to prepare a paper upon the 'Differential Diagnosis between Dengue and Yellow Fever, with Some Account of the Epidemic in Texas in 1897,' to be read at the forthcoming meeting of the State Medical Association at Houston, and at the Denver meeting of the American Medical Association. If dengue prevailed in your community during the last season, you would oblige me very much by furnishing me with information upon the following points:

"1. Date of outbreak; rapidity of spread; probable proportion of population affected; duration of epidemic; any evidence as to its origin.

"2. Any unusual meteorologic influences, as excessive heat or precipitation; local hygienic conditions.

"3. Symptomotology: *a.* The eruption: The character of, and present in what proportion of cases. *b.* Glandular enlargements: What glands usually involved, and in what proportion of cases. *c.* Hemorrhages: In what proportion of cases and from what sources. *d.* Nausea and vomiting: Severity and comparative frequency of; dysenteric symptoms noted. *e.* Albuminuria: In how many instances examined for, proportion of cases found, large or small amount; any other characteristics of urine noted, daily quantity, color casts, blood, epithelial cells; any uræmic symptoms or other evidence of acute nephritis.

"4. Ratio of pulse to temperature.

"5. Jaundice, if noted, character of, and occurring in how many cases.

"6. Mortality, postmortem findings, if any.

"7. Did you have any reason to suspect the presence of yellow fever? If so, upon what evidence.

"8. Any difference in the severity as the epidemic progressed?

"As I am sending out but a limited number of these circulars, and the subject is of extreme interest and importance, a prompt and complete reply is requested."

Data was obtained as follows: Gainesville, Cook County, on the extreme north central border, reporter, Dr. J. E. Gilcreest, no epidemic, only a few sporadic cases of mild type, symptoms not noted. Terrell, Kaufman County, near northeast border; Dr. W. H. Monday reports no cases. Dr. J. T. Wilson, Sherman, Grayson County, north central border, no dengue. Dr. J. M. Neil, Bonham, Fannin County, in same district as above, no epidemic; saw but one case, which was contracted at Paris. Dr. T. J. Bell reportsfrom Tyler, Smith County, East Central Texas, very mild epidemic, commencing 15th of October, spreading slowly and affecting a small number, no evidence as to origin, no rash in most cases; no hemorrhages, glandular, involvement, dysenteric symptoms or jaundice noted; no deaths.

Dr. J. H. Barham, Nacogdoches, East Central; no epidemic, saw three cases only; one contracted dengue in Houston, two here, all in different families, no spread, eruptions in two, absent in one, nausea and vomiting in all; no test for albumin; jaundice in all, well marked in one, moderate in one, slight in the other; suspected yellow fever in the case from Houston; all recovered.

Summarizing results obtained from the foregoing twenty observers in eighteen places, we find as follows:

The epidemic of dengue of 1897 prevailed chiefly in Southern Central Texas within a radius of 200 miles of Houston, but extending and prevalent to a limited extent 300 miles to the extreme northern border. San Antonio, Houston and Galveston, followed by Schulenburg and Navasota, appear to have been most intensely infected, the proportion of population in these places affected varying from 75 to 90 per cent. Belton, Palestine and Huntsville followed closely with a percentage of 60-65. In the extreme Northern and Eastern portions of the State, as in Gainesville, Sherman, Bonham, Terrell and Tyler, there were but a few sporadic cases. At Dallas and Paris a very mild epidemic, affecting not more than 6 per cent. of the population. San Antonio, Galveston and Houston seem to have been the centers of infection, as appears from the following statement. Palestine, a railroad center about 150 miles north of Houston and 200 miles northeast of San Antonio, with double daily train service. Dr Link states the probable origin was from these two cities.

Dr. Tabor reports from Bryan, which is about 100 miles from Houston with direct rail connection, that both the first and second cases were persons who had been exposed in Houston. Dr. Thomason reports from Huntsville, about seventy miles north of Houston, with direct train service with the latter place and Galveston, that the first case was a young lady from Galveston, and that other foci of infection were soon developed by parties from Houston and Galveston. Austin's first case was a hotel clerk recently returned from San Antonio. Dr. Lee reports from Galveston that his first case, seen July 29, was from San Antonio.

The first two cases seen by the writer, August 11, were in the persons of young men who had returned to Galveston from the Interstate drill at San Antonio. Schulenberg is about equidistant from Houston and San Antonio, and may have been infected from either. Navasota is only about seventy miles from Houston. The intensity of the disease at San Antonio, the presence of the militia companies, not only from numerous points in the State, but of States east of the Mississippi, the coincidence in time (latter part of July) of the return of the militia to their homes with the outbreak of the disease, and the positive identification of the first cases in several places in persons who had returned from the encampment, is evidence going to prove that San Antonio was the initial point of infection in the State. As to the origin of the disease in the latter city I have no means of tracing it, but can only say that ample opportunity for conveyance of dengue was afforded by the collection there from July 19 to 26 of troops and visitors from points east of the Mississippi where the disease had made its appearance.

Further confirmation is also afforded by the facts above cited that dengue is an infectious, a portable and contagious disease, *i. e.*, the specific micro-organism which produces it may escape from the body of an infected individual and be transmitted by persons and things to other persons, thus speedily multiplying foci of infection and accounting for the rapidity of its dissemination. The fact previously observed that the progress of the disease is arrested by the advent of cold weather or frost, has also been confirmed by the consensus of statements herein presented. As to the effects of meteorologic and hygienic conditions, the majority of observers describe the weather as excessively hot and dry. The mean daily temperature in Galveston was nearly 8 degrees hotter during the month of October, 1897, than in the previous three years, and the continuance of unusual hot weather in November and December was the subject of general comment. Local sanitary conditions do not appear to have played any important part in the propagation of the disease. Dr. Paschal, however, calls attention to the excessively insanitary condition of San Antonio at the time of the outbreak, and Dr. Tabor ascribes the comparative immunity in Bryan (only 100 cases out of a population of 4000) to the extraordinary precaution to better the sanitary condition of the town. In regard to the symptomatology, I have not attempted to obtain information as to the complete clinical history of the cases under consideration, but rather to determine the distinctive value of those symptoms which have heretofore been regarded as of great importance in the differentiation between dengue and yellow fever, viz., the presence of an eruption, hemorrhages, nausea and vomiting, evidence of an acute nephritis, as albuminuria, scantiness, presence of casts, uremia, divergent pulse and temperature, jaundice and mortality.

There is almost perfect unanimity upon the part of observers as to the presence of an eruption, the proportion with this symptom varying from 25 to 90 per cent; 75 per cent would be a fair average. It is variously described as miliary, scarlatini-form, rubeoloid, erythematous, reddish papules, macular dermatitis, urticarial desqunamation, as localized upon the face and neck, upon the face and upper part of the body, with profuse perspiration, irregular as to time of appearance, coming on the second or fourth day. or end of the fever, hyperemia appearing on the first day. Dr. Peeples notes the fact that, while frequently absent in adults, an eruption was almost invariably present in children.

The consensus of opinion is that glandular involvement occurred to a very limited extent in this epidemic; the majority of observers not noting this symptom at all, but one observer mentioning enlargement of the cervical glands and tonsils in 25 per cent of the cases; another the connection between the enlargement of these glands and the rash; another observed swelling of parotids, sublingual, inguinal and suboccipital, especially in children; one observer noted enlargement of the submaxillary glands in 20 per cent of his cases.

*Hemorrhages.*—With but three exceptions every observer noted the occurrence of hemorrhages. The nose, throat, gums, stomach, intestines, uterus and kidneys were the chief sources mentioned. The percentage in which this system was present varied from 1 to 10 per cent. Three or four observed black vomit.

*Nausea and vomiting* were notable symptoms and occurred in a large proportion of cases, varying from 80 to 90 per cent; with but few exceptions it was described as severe, excessive, incessant, almost universal, etc.

*Condition of the Urine.*—Unfortunately, observations pertaining to the urine were made so infrequently and imperfectly as to render them of but little value. Most of the observers made no examination at all, a few examined for albumin only in the worst cases. Dr. Wilkinson, of Galveston, out of 500 cases, says he tested for albumin in many and found it absent; sp. grav. was generally high. Dr. Lee, Galveston, states he examined for albumin in fifty cases out of 490. Eight of these had previous kidney trouble; of the remaining forty-two, twenty showed slight albuminuria on the third and fourth days; and in six albumin was present in large amounts. Dr. J. M. Coble, Dallas, examined the urine in 25 per cent of his cases, and found slight albuminuria in 10 per cent of them.

Drs. Wilson and Harris, of Navasota, only made urinary examinations toward end of the epidemic, in about fifty cases; in forty of these albumin was found in good quantity. The urine was diminished in quality and high colored; casts and blood cells found in three cases, and uremic symptoms in four. Dr. Taylor Hudson, Belton, tested the urine only in a few suspicious cases, and found albumin in several; the urine was usually dark and diminished; uremic symptoms pronounced in several. Dr. D. L. Peeples, Navasota, found albumin from slight trace to abundance, in some cases with almost total suppression, color varying from a deep straw to color of blood. Dr. R. R. Walker, Paris, found albumin in 25 per cent of his cases. Dr. W. W. Walker, Schulenberg, only tested for albumin in worst cases; found it in ten, in some in large amounts. The remainder of reporters failed to note condition of the urine.

If, however, dengue was the only disease prevalent, albuminuria is not the rare symptom in that disease, as heretofore has been the opinion.

*Ratio of Pulse to Temperature.*—Ten out of sixteen observers mention that the pulse was abnormally slow in proportion to the pyrexia; three state the ratio normal, and three made no note of the symptom.

*Jaundice.*—Four reporters mention this symptom as present in about 7 per cent; one, Dr. Wilkinson, Galveston, mentions jaundice as being of frequent occurrence and of severe grade after October 1. One notes it as generally present of mild grade; another states it was mild in 10 per cent and severe in 2 to 5 per cent; one found it more or less present, and seven failed to note icterus at all.

*Mortality.*—There is almost perfect unanimity as to want of mortality in the recent epidemic, the few deaths occurring were usually ascribed to some complication. As regards progressive severity a majority state there was no marked difference; a few assert aggravation in the beginning and in the midst of the epidemic. Dr. Wilkinson notes mildness in August, increased severity in September, progressing to violence in October. Only two or three out of the twenty suspected the presence of yellow fever. As to the early symptoms: The onset was usually sudden, attended by slight rigors or chilly sensations with more or less severe headache and pains in the back, limbs and joints. As regards the paroxysm there is a remarkable discrepancy in the statements of Drs. Lee and Wilkinson, each of whom treated about five hundred cases. Dr. W. (I quote his exact language) states: "The fever in all my observations contained but one single paroxysm; there was no secondary fever from first to last; it departed by lisis, three or four days duration and never returned, except in cases of relapse several weeks later." Dr. Lee, on the contrary, says: "In a large proportion of the above mentioned cases two paroxysms were noticeable, the first coming usually without positive chill or chilliness; temperature usually high in the beginning became, after slight remission, high again toward the end of the attack."

It is difficult to reconcile these contradictions. The probabilities are that there is no invariable rule in regard to the paroxysm. In ten cases at the Sealy hospital where careful thermometric observations were made, there was one paroxysm only in seven; in one there was an exacerbation in the afternoon of the day following that, when the temperature reached normal; in one the fever was of remittent type, rising at midday for three successive days; in one there was a slight return of fever on the evening of the first day after the temperature reached normal.

Before proceeding to make any deductions from the foregoing, it will be instructive to present a brief review as to the statements of authorities upon the differential diagnosis of the two diseases under consideration. An authority upon this subject I understand to be one who has had the opportunity of studying them at the bedside. I shall not attempt any complete bibliography, but the following I think will show what has been the accepted teaching:

H. D. Schmidt on Dengue, Pepper's System of Med. Ed. 1885, pages 884-885, says the temperature steadily rises in yellow fever, it is remittent in dengue; pulse in yellow fever falls on third day, while the temperature continues to rise, in dengue on the contrary, the pulse rises with the temperature. In the condition of the stomach also dengue considerably differs from yellow fever, for while in the latter disease this organ is almost always irritable, and vomiting frepuently present, it is but rarely affected in dengue. The urine in yellow fever very frequently contains albumin as soon as the third day, in dengue almost never. Finally the absence of jaundice and the appearance of the eruption on the fourth or fifth day removes all doubt about the nature of the disease.

Sternberg on Yellow Fever, American System of Med., Vol. 1, page 290: "If a diagnosis has not been made before, the symptoms which characterize the second period of the disease should serve to differentiate it from all forms of malarial fevers. The cool and usually moist skin, the abnormally slow soft pulse, the gastric distress, and pain on pressure in the epigastrium, the yellow tinge of the conjunctiva, and the albuminous urine in a patient whose temperature is normal or subnormal, following a febrile paroxysm as represented in charts, certainly furnishes a clinical picture which should be recognized." Speaking of dengue, pages 291-92, he says: "It also is characterized by a febrile stage of comparatively short duration, but the characteristic symptoms of yellow fever are absent. Albuminous urine, stage of depression, hemorrhages, etc.

Matas, Keating's Cyclopedia, Vol. 1, page 894, quotes Holliday, who makes a comparison of the symptoms of the two diseases after a careful analytical study of the opinions of over sixty physicians who had a long experience of both diseases in New Orleans and other parts of Louisiana, as follows:

| YELLOW FEVER. | DENGUE. |
|---|---|
| Temperature rising regularly. | Temperature rising irregularly. |
| Tongue white center, red edges pointed. | Tongue broad white indented edges, rarely red. |
| Stomach irritable, vomiting frequent. | Vomiting rare. |
| Conjunctiva congested, jaundice appearing early. | Conjunctivae rarely red, jaundice never observed. |
| Secretions all suffering, urine scanty, often albuminous, suppression frequent. | Secretions natural, urine usually normal, sometimes exceptionally traces of albumin. |
| Hemorrhages frequent and alarming, black vomit an urgent symptom. | Hemorrhages slight, black vomit very rare. |

In regard to the condition of the urine in dengue, Matas observes (see foot note, page 889, Keating) "that in tropical pyrexiæ it is always a matter of great importance, both from the diagnostic and prognostic standpoint." The earliest observers of dengue satisfy themselves with noting simply the color, quantity and reaction, and these are usually most diversely described. Other observers are, however, much more satisfactory. Thus Morgan noticed a specific gravity 1004 to 1040, acid, *non-albuminous;* Chipperfield, acid, sp. grav. av. 1010, *non-albuminous.* Goodeve detected an occasional trace of albumin in four cases in the Indian epidemic in 1853, while Charles and Martialis *never detected* it in the epidemic of 1872. In China at Amoy, Muller and Manson *failed to find albumin.* Albuminuria was detected *only once* in the epidemic of Martinique in 1860, and twice in Cochin China by French observers in 1875 (Mahe). Albuminuria was observed exceptionally by Holliday and his co-laborers (loc. cit.) in Louisiana. Enough has been said to prove that albuminous urine is an exceptional occurrence in dengue, which differentiates it markedly in this respect from yellow fever.

Eugene Foster, Ref. Handbook Med. Sciences, Vol. 2, page 397, gives the following summary of the points of similarity and dissimilarity in dengue and yellow fever.

"In time of appearance, and generally in geographical distribution they seem related to one another. Dengue has, however, prevailed in Asia and Egypt, where yellow fever is unknown. Both diseases are arrested by severe frosts. Both dengue and yellow fever are diseases characterized by one febrile paroxysm." In order to show contrast, the symptoms will be arranged in parallel columns:

| YELLOW FEVER. | DENGUE. |
|---|---|
| The fever rises steadily. | The fever rises regularly until the acme is reached, when a short stadium of a few hours occurs, followed by a remission, when a second rise of temperature takes place, but not reaching the former height. |
| The pulse becomes slower, while the temperature rises. | The pulse increases in frequency with the rise of temperature. |
| Fever lasts seventy-two hours. | Fever lasts five to eight days. |
| Vomiting frequent. | Vomiting rare. |
| Eruption rare. | Eruption common. |
| Jaundice almost invariably present. | Jaundice extremely rare. |
| Urine scanty, frequently albuminous and often suppressed. | Urine generally high colored, normal in quantity, free from albumin and never suppressed. |
| Hemorrhages frequent, alarming and often fatal. | Tendency to hemorrhages from nose, gums, bowels, lungs and womb, with occasional black vomit, but hemorrhages, as a rule, insignificant. |
| Often fatal. | Proverbially non-fatal. |
| One attack protects from another. | One attack not protective against another. |
| Not protective against dengue. | Not protective against yellow fever. |

Quoting Sternberg again from his article in Buck's Reference Handbook, Vol. VIII, page 60, speaking of the diagnosis of yellow fever, he says: "Of the three prominent features making up the clinical tableau of yellow fever, viz., a yellow skin, highly albuminous urine and black vomit, only one is a constant character which can serve in establishing the diagnosis in mild cases; this is the presence of albumin in the urine. At some period in the disease, even in the mildest cases, there will be a distinct trace of albumin in the urine as shown by the usual tests, and this will be usually sufficiently abundant to leave no doubt in the mind of the observer as to the nature of the precipitate. The value of this test in the differential diagnosis is indisputable. It is true that a trace of albumin is sometimes found in the urine of severe cases of fevers of malarial origin, but in cases of yellow fever of equal severity as compared with these, the precipitate would, as a rule, be very abundant on the third or fourth day of sickness, forming a deposit to the extent of one-fourth to one-half of the contents of the test tube, or even more. At the same time a microscopic examination would show the presence in the urine of numerous granular casts from the tubuli uriniferi. These are found also, although in a smaller number, in the urine of the milder cases during the second stage of the disease.

"The second stage of the disease is commonly, however, well marked in non-fatal cases, and is its most characteristic feature, the remarkably slow and soft pulse, the evident prostration of the vital powers, although the patient may be comfortable, and even cheerful and desirous of getting up and taking food, the yellow tinge of conjunctivæ and skin (not always present), the tenderness on pressure in the epigastric region, and often a feeling of weight and distress, attended with intense thirst and vomiting of a transparent acid fluid, or of the characteristic black vomit, the tendency to passive hemorrhages from the mucous surface of the mouth or nose, oozing of dark blood from the gums or lips, or sides of the red and fissured tongue; the scanty urinary secretion, and the presence of albumin, usually in considerable amount, constitutes an unmistakable ensemble of symptoms."

It appears from the above quotations that the symptoms which have heretofore been relied upon to differentiate between yellow fever and dengue are the occurrence in the former of albuminuria, the characteristic facies (inclusive of jaundice), the divergent pulse and temperature, excessive irritability of the stomach, and increased disposition to hemorrhages. The absence of such symptoms in the main, the presence of an eruption in a large proportion of cases, and a want of mortality are characteristic of dengue. In the epidemic herein described we have an apparently inextricable confusion. A widespread epidemic of fever presenting the symptomatology of yellow fever in many instances, but with the eruption of dengue and practically with no mortality. One of two deductions is irresistible, either the two diseases approximate more intimately in their symptomatology than has heretofore been taught or yellow fever of remarkably mild type has been associated to a greater or less degree with dengue; granting the reliability of the testimony herein presented, both conclusions are warrantable. I shall not attempt here to solve this problem so far as the epidemic of the interior is concerned, but will present the evidence which is convincing to my mind that the latter proposition is true as to the coast cities of Galveston and Houston. Before doing so, however, let me refer briefly to certain facts in regard to the epidemic east of the Mississippi taken chiefly from the report of the Louisiana State Board of Health, dated December 6, 1897. The first death from yellow fever was reported by Dr. J. M. Holloway, of Louisville, Ky., about August 18th, the patient having gone to that city from New Orleans via Ocean Springs, where the disease was supposed to have been contracted. Subsequently to which, four official investigations were made as to the nature of the epidemic at Ocean Springs, resulting as follows:

1. The visit of Dr. S. R. Oliphant, president of the Louisiana Board of Health August 22, 1897, who reports finding an epidemic had been prevailing the previous six weeks, that 400 cases had occurred in the practice of two physicians, without a single resultant death, and that it was considered to be dengue of mild type.

2. On August 23, a commission of experts from the New Orleans Board, from the Mississippi State Board and others signed a report which concludes as follows: "After a careful inspection and examination of the aforesaid cases, we are positive in our opinion that the disease is dengue, and that in no case is there or has there been any symptoms which would lead to even a suspicion of a more serious disease."

3. On August 27, we have a report of another commission, to which the name of the health officer of Alabama is added, stating: "In reply to your request we have again investigated the fever at Ocean Springs, which is abating, and absolutely without fatality. The conclusion arrived at was *that it is not yellow fever.*"

4. On September 6, we have the following satement: "The patient died Sunday night, and early Monday morning the expected autopsy was performed by the bacteriologist of the board in the presence of the medical gentlemen assembled. Unmistakable evidences of yellow fever having been revealed, we arrived at a unanimous verdict."

According to Dr. Oliphant the grand total of cases in New Orleans did not reach 2000, nor the deaths 300, a mortality of 15 per cent based upon reported cases which would doubtless be much smaller if founded upon actual number. Another significant fact noted by Dr. Oliphant in the epidemic, which verifies the history of previous ones in New Orleans and elsewhere, is that yellow fever does not burst forth suddenly, but gathers volume and force after smoldering for a time, e. g. it was introduced into Edwards, Miss.,

August 8, but gained no headway until the middle of September. Confirmatory as to the remarkable mildness of the last epidemic* Dr. L. Sexton writes from New Orleans September 20, "*Our present death rate is lower than in any epidemic of yellow fever, if the* present death rate prevails throughout this visitation we will have to send around a subscription list for our undertakers."

What bearing have these facts upon conditions as they existed in Texas? In my opinion they are convincing when taken in connection with the clinical history of certain cases that the epidemic upon the Texas Gulf coast was similar to that in Louisiana, Mississippi and Alabama, the results being modified by the late introduction of yellow fever infection and by the presence of unfavorable conditions for its dissemination. It should be remembered that the declaration of quarantine by Galveston was made on September 10, nearly a month after the recognition of yellow fever at Ocean Springs, that the latter place is directly upon a common route of travel between eastern and Texas points, and that a month subsequently the diagnosis of yellow fever in Galveston and Houston was made by Dr. Guiteras. Under such circumstances it is not surprising that yellow fever infection should have been introduced into Texas; nor should it be a matter of wonderment that it should not have been generally recognized when we take into consideration that it came in the guise of dengue, was introduced late in the season, into localities where the conditions were unfavorable for its spread, and that according to all testimony, it was the mildest epidemic of yellow fever ever known in the history of the country.

Admitting that there is greater similarity in the symptomatology of the two diseases than has heretofore been acknowledged, the question arises, how can they be differentiated? In my opinion chiefly by the symptom complex of an acute nephritis in yellow fever and its absence in dengue. In the latter, simple parenchymatous changes may occur in the kidneys and be manifested by an evanescent and mild albuminuria. While in the former in a series of cases many will afford incontestible evidence of the occurrence of a severe nephritis, viz., scanty urine, of high color and specific gravity, intense and persistent albuminuria, hematuria, casts, decided tendency to suppression and the accompanying uremia.

The following are brief clinical histories of the three cases, two reported by Dr. R. T. Morris occurring in Houston* and one from Galveston reported by the writer:

---

*American Medico-Surg. Bulletin, Oct. 10, 1897, page 903.

*S. W. Med. Record, January, 1898, p. 415.

Case 1.—Oct. 23, N. J., aged 28, was called late in the evening; found patient suffering with anorexia, pain all over the body, but especially marked below the knees; constipated but urinating freely; temperature 103; severe frontal headache; no albumin in urine. Prescribed castor oil, and being called out of the city, did not see the patient till three bays later, when he was found to be very low; pulse hardly perceptible, not over fifty-four to the minute; retching but no vomiting; urine heavily loaded with albumin; total amount voided in twenty-four hours, three pints; patient muttering to himself, but on speaking loud to him could be aroused, and would give intelligent answer, although a few minutes after conversation ceased he would drop back to unconsciousness; at 6 p. m. vomiting of a coffee-ground-like substance set in, not more than a tablespoonful every hour till next morning, when, of a sudden, without any effort whatever, a gush of same kind of vomit was ejected, amounting to at least a pint and a half. Inside of fifteen minutes after this vomit patient expired, and after ten minutes his corpse was of a decided yellow tinge, and dependent portions discolored like severe bruising.

Case 2.—September 19, called to see I. R. F., male, aged 22, found what I believed to be incipient dengue; reported improved by the 21st; on the 22d recalled in haste; on arrival found patient comatosed, pulse 56, temperature 103.5 degrees; on arousing he told me, and the statement was confirmed by his mother, that all urine he had passed since the day before (in all 28 hours) was in a vessel under the bed. By ocular inspection was found to be very dark and muddy, smell strongly ammoniacal, examined at office, albumin in great quantity, nearly solidifying in test tube. Returned at once to patient; found him again in comatose condition and having voided a small quantity of urine which had strongly colored the linen. Prescribed tincture digitalis, ergot, spirits juniper and liquor ammonae acetatis. Recovery uneventful but exceedingly slow; yellow tinge in eye appeared as late as 26th, disappearing after forty-eight hours.

Case 3.—R. T. Jr., native of Galveston, aged 16, of robust constitution and previous perfect health, was taken sick suddenly October 24, in the afternoon, with a slight chill followed by high fever, with intense headache, pains in back, limbs and joints. At my first visit on the 25th, as soon as the room was entered a peculiar nauseating odor was perceived, the eyes were watery and red with a yellowish tinge, the skin was decidedly yellow, the gums were softened and disposed slightly to bleed, anorexia was marked, tongue coated and red at its tip and edges, excessive nausea and disposition to vomit everything swallowed, bowels had moved from a purgative. The patient was nervous and disposed to sleep. The course of the fever is typical and is exhibited in the accompanying chart. It should be noted that when a normal temperature was reached on the 28th, the fifth day of the fever, that the pulse dropped to 64, and that on the 30th when the temperature was subnormal the pulse was 50. The stomach continued to be irritable, there was epigastric tenderness. On the night of the 29th blood was vomited in small quantity; the urine was scanty, of high color and specific gravity. A heavy precipitate of albumin was found on the 29th, also granular casts. Daily examinations showed albuminuria during the following eight days, after which it gradually subsided as the urine became paler and more abundant. The albuminuria was more intense during the stage of depression. After a tedious convalescence recovery ensued. My diagnosis of a severe attack of yellow fever was confirmed by Drs. McLaughlin and Magruder, both of whom have had experience with this disease.

It is useless to add any comments or summarize any other cases with similar histories. What other diagnosis could have been made than that of yellow fever. *Anamolous dengue* was one of the names suggested during the recent epidemic; if this is dengue, then the identity of that disease and yellow fever is established. When pressed for a diagnosis in certain of these cases, some of the Galveston experts denominated them Weil's disease, or acute infectious jaundice, which is manifestly such a far fetched conclusion that words would be wasted in its refutation.

One of the arguments which has been repeatedly used against the existence of yellow fever in Texas during the past season, was the want of mortality. Upon this point let me quote Dr. Morris again:* "From October 1, 1897, to November 18, the death rate in Houston was 33 1-3 per cent greater than in the corresponding period of 1896." The causes given in death certificates are as follows: senility 11 (four of whom were not over 60); dengue 5; enteritis and gastritis 11; fever 10; meningitis 1; kidney disease 5. The record of Galveston does not show any increase of deaths during the months of August, September and October over the previous three years. There were five deaths, however, preceded by symptoms of yellow fever; it is a significant circumstance that in four of these uremia was given in the certificate as the cause of death. In the other, congestion of the lungs was mentioned as the primary, and bilious fever as the remote cause.

Those who deny the existence of yellow fever in Texas during the past season rely chiefly upon the apparent indisposition of the disease to spread from numerous foci. In other words, they contend that the presence of the infection necessarily involves its dissemination. No extended argument is requisite to demonstrate the fal-

*S. W. Med. Record, January, pp. 413-414.

lacy of such conclusions. The pathogenic micro-organism of yellow fever is a facultative parasite, but its ordinary mode of life is saprophytic. It grows and develops outside of the body when the conditions are favorable for its reproduction. The seed may fall by the wayside, or upon a rock, or among thorns and wither away, or upon good ground and spring up and bear a hundred fold. The fact that the soil of Texas was an unfavorable one for the multiplication of the yellow fever germ last season is an adequate explanation of the history of the recent epidemic. It is not contended that the hygienic condition of these cities was perfect, but that the combination of circumstances was antagonistic to the extensive dispersion of the infection. Instead of being a blot upon the sanitary escutcheon of Houston and Galveston, it is another demonstration of the fact which has previously been observed, that yellow fever may be brought to these places without necessarily involving an extensive epidemic or serious mortality. Two important lessons should be emphasized; 1, the urgency of promptly recognizing the early cases and calling them by their proper name, for by this means only may subsequent disaster in many instances be averted; 2, we should put our houses in order, in other words, adopt every possible method of domiciliary and municipal cleanliness. Napoleon is said to have remarked that "Providence was on the side of the strongest artillery." When yellow fever is around Providence is on the side of the town with the best system of sewers. Again, it has been demonstrated that yellow fever may masquerade in the garb of dengue and that the latter is a portable disease; leaving out of the case the probable association with yellow fever, it became a serious question whether dengue should not be considered a quarantinable disease. The suffering and expense incident to an epidemic like the one of last season would certainly appear to justify measures of prevention.

DISCUSSION.

Dr. Geo. H. Lee: I desire to discuss the paper of Dr. West. I commend it as admirable, clear, forcible, conservative, fair. It is to be regretted that it is not more comprehensive. Reports from more practitioners and from a larger section of the State would have added to its value.

With some of his conclusions I am compelled to differ. The paper leads its author to a position which is unavoidable; that the symptoms relied upon in the past by the authorities to differentiate yellow fever have been conclusively shown to occur in dengue, and did appear in dengue in San Antonio, Houston, Galveston, Austin, Palestine, Belton and other points in Texas during August, September, October and November, 1897. This conclusion would have been strengthened by reference to the epidemic of dengue in Texas in 1885, in which the same facts were noted, at a time when there was no suspicion of presence of infection of yellow fever. I will read a note from Dr. Paine relating to his observations upon these points:*

*New Orleans Medical and Surgical Journal, January, 1898.

Galveston, November 20, 1897.

My Dear Doctor Lee: Your faver of the 12th inst. with reference to my experience on certain points connected with the epidemic of dengue which prevailed here in 1885 came duly to hand, but the pressing demands of my occupations have caused me, unintentionally, to overlook it until now, for which omission I beg you will forgive me.

I had frequent occasion to examine the urine of patients suffering from dengue during the prevalence of the disease in this city in 1885, and found albumen, in varying proportions, in numerous instances. Similar observations have been noted by other physicians of this place. In the light of recent events, I greatly regret that I did not examine the urine of every patient so affected as a matter of routine. The record of such an invetigation would doubtless have possessed inestimable diagnostic value. The want of correlation between pulse and temperature was a noticeable feature in the majority of cases who had the disease in severe form. Very sincerely yours,

(Signed) J. F. Y. Paine.

In his remarks before the Ninth International Medical Congress* he also referred to the frequent and severe hemorrhages in these cases.

This conclusion is just the consideration which made me doubt the diagnosis of Dr. Guiteras on October 9th, in Galveston. No commercialism, no passion, no prejudice influenced my conviction.

I had been treating dengue fever over thirty years. Was sick from October 2nd to 9th. And on emerging from sick room to find that Dr. Guiteras had announced the presence in Galveston of eight cases of yellow fever, I naturally concluded that the infection had crept into the city, and I applied to Dr. Guiteras expecting to hear from him the new features which had appeared. To my surprise, I learned nothing new. The same features he relied upon I had seen for a month, long enough for an epidemic to incubate, and with a city full of sick people, thousands of cases of the prevailing disease, the mortuary report showed a mortality of 7 per 1000 for the year, and, as far as I knew, no mortality from the prevailing sickness.

Under such circumstances I felt justified in hesitating to accept a diagnosis based upon such, to me, insufficient grounds.

The past epidemic, as Dr. West's paper shows, further points to necessity for revision of prevailing statements regarding the importance and value of certain symptoms as differentiating yellow fever from dengue.

Hemorrhages, divergence of pulse and temperature, jaundice, albuminuria, have been shown to occur in both diseases,—the difference being rather of degree than of kind.

Dr. West impresses the importance of the presence of acute nephritis of severe type as a distinguishing feature of yellow fever. Here he moves in a circle,—returning to the original position of Dr. Guiteras, who was evidently misled by the presence of albumen in the urine.

If we concede that a mild nephritis does occur in a fair proportion of dengue fever patients can we decline to accept that this mild nephritis may become a severe type in occasional cases where the individual's condition is influenced by constitutional or other causes?

Of the records of urine examinations preserved in my notes, more than 10 per cent show nephritis accompanied by a large amount of albumen. These cases with one exception had well marked rash,— in the exceptional case slight desquamation occurred. These cases had other well marked dengue symptoms. No black vomit. No marked hemorrhages. All recovered.

Can we rely on any one feature as diagnostic? The doctor mentions several hypotheses as the result of various features of this epidemic.

It is my purpose to discuss these briefly, and I shall consider first numbers one and two together.

"The disease was only dengue fever. Anomalous cases presented much of the symptomatology of yellow fever, but not proven to be of that nature, because failing to spread from foci, and unattended by mortality."

To my mind these hypotheses are reasonably true.

Following a suggestion taken from a paper of Dr. Scott, I have tried to find one instance in which some individual had suffered from both diseases. I have been unable to hear of such an instance. Among my own patients, three had one relapse, and one man had three well defined attacks, the last in early November. Yet there was nothing in the clinical history of either case that could be regarded as suspicious.

Surely if Galveston had had fifty and Houston one hundred cases of yellow

---

*Transactions, Ninth International Medical Congress (1887), Vol. 14, pp. 470-1.

fever in an epidemic of thousands of cases of dengue, such instances would have been noted frequently.

Regarding the spread from foci: In our city we had at least thirty cases, each unassociated with any other, diagnosed as suspicious and yellow fever, and absolutely no new case from any of these foci, except in the instance of the man named Wade who died at the Sealy Hospital. Following this case, the interne who saw him and one of the physicians were ill with infections that were regarded as suspicious. It was remarkable what a malignant influence the fine sea air of Galveston had upon the infection. When Dr. Guiteras left there were 13 cases in the city. After his departure it was nearly three weeks before another case was diagnosed. I am aware that Sternberg holds the period of incubation of an epidemic is from two to six weeks but bear in mind that the first case which Dr. Guiteras referred to as suspicious died on September 9th, and that each one of these thirteen cases present on October 10th represented an independent focus of infection.

Regarding the mortality: I think after careful search that ten will cover the number of deaths that might be in any manner attributed to dengue or suspicious illness in Galveston during those three months, August, September, October. The mortality from all causes was 23 less than same period in 1896, and 31 less than in 1895. We must have had 30,000 cases of the prevailing sickness.

I must object to accepting Dr. Morris' arguments upon the mortuary reports in Houston unless he has traced out every case and can state positively this death was suspicious. To illustrate my objection, I refer to the report of Dr. Guiteras to the Surgeon General of the M. H. S. in which he mentions as occuring in the mortuary reports of Galveston for September, three cases in which the death was probably from yellow fever. In each of these instances* I was able to show clearly that there was no cause for suspicion and I am sure the doctor would not have included either in the manner he did if he had first investigated each case.

Passing on to Dr. West's hypotheses: I shall not discuss the suggestion that the epidemic was yellow fever only. No one believes so.

Hypothesis No. 3. "During the epidemic, yellow fever apeared in mild form in Galveston and Houston and was unrecognized." Or putting the proposition in a little different form: During the epidemic of dengue, a limited number of cases occurred in no way associated with or traceable to each other, of a more aggravated type, in which the symptoms strikingly resembled yellow fever, and regarding the nature of which there is a difference of opinion.

Among my own patients a number were very sick, displaying in varying degrees the symptomatology formerly regarded as indicating yellow fever. In six, large amount of albumen was found in the urine on the third and fourth day. Only one subnormal temperature was noted. No case in which a stage of depression developed. The yellowness, though mild, was distinctly an icterus, as contrasted with the saffron yellow accompanying superficial turgescence described in yellow fever, the result of staining from blood pigments. I had no suspicious case and only one death. The detailed history of which is given in the issue of the N. O. Medical and Surgical Journal referred to. The patient died of chronic nephritis complicating dengue.

Now if the cases (by some considered yellow fever and by others anomalous dengue) were yellow fever, where did the infection come from?

Dr. West says in his paper that the infection was introduced as dengue. Well, dengue was in Texas in middle July. Dr. Guiteras located the first suspicious case as dying September 9th. If introduced thus in midsummer, why does it die out and disappear with a mortality of nil, when a like condition of affairs in New Orleans produces 3000 or more cases and a mortality of fifteen per cent?

Dr. West quotes the parable of the sower, which reads beautifully but does not explain.

If this was the infection of yellow vfeer, how does it happen that it apears in scattered foci in Houston and Galveston, also in other places of the State, preceded and folowed by typical dengue?

If Dr. Guiteras was not in error when he declared that the infection was in both places in scattered foci, and would become epidemic, how did it happen that the course of subsequent events failed to develop the epidemic he anticipated,—as I am informed, the only instance in which he had diagnosed an epidemic status that did not materialize.

The fact is it has never been shown that the infection of yellow fever was ever in Texas in 1897.

---

*New Orleans Medical and Surgical Journal for December, 1897.

If you refer to the epidemic as it began in Ocean Springs, you find that the clinical history first produced suspicion, but the autopsy made the diagnosis. And if you read the notes of that autopsy you can not question the diagnosis.

Now we had autopsies in Texas on cases considered suspicious or diagnosed ante-mortem as yellow fever, but not in a single instance do the notes of the pathologist correspond to the findings in the case at Ocean Springs and the Gelpi case in New Orleans.

My friend Dr. West is disposed to poke a little fun, if you will pardon the expression, at Drs. Burke and Wilkinson in connection with the Wade case which they called Weil's disease or acute infectious jaundice.

Let me read you portions of the report of the autopsy.

"The body was that of a male, aged about fifty years, and weighing about 120 pounds.

"Inspection showed marked emaciation. The skin was golden yellow on body and extremities; face and neck reddish yellow. Cadaveric rigidity well marked. Crusts of dried red blood were observed around the mouth and rectal orifice, the result of previous hemorrhages. In the lumbar and axillary region were perforations of the skin, not bleeding (the result of hypodermic injections during life) and some small red ecchymotic spots on parts of the body subject to pressure. On section, the thoracic and abdominal walls, showed little fat. Lungs, normal. Heart, normal in size, and fat around same golden yellow. Liver, considerably enlarged, congested. Section followed by oozing of dark blood from cut surface. No boxwood appearance or oozing of oil globules or other evidence of yellow hypertrophy. Gall bladder, enlarged and filled with straw colored fluid, stomach filled with gray-black fluid, the result of the action of tincture of iron upon its contents, with odor of whisky. Mucous membrane, congested. No punctate hemorrhages. Small intestines, normal; contents gray, black and plastic, consistence of cream. Large intestines, normal; contents gray, black and plastic, with fecal odor. Bladder, empty and firmly contracted. Kidneys, left contracted; weight, 6 ounces 135 grains;—right swollen, smooth; weight, 7 ounces 330 grains. Spleen small, firm; weight, 4 ounces 220 grains.

"Microscopic investigation by Dr. Allen J. Smith, professor of pathology, was as follows:

" 'Pathological Laboratory, University of Texas.

" 'There were presented to the Pathological Laboratory of the University of Texas from the case of Mr. Wade, of Sealy Hospital, the liver, kidneys and spleen. Of these the liver and left kidney have, to date, been examined; other tissues are in course of preparation. The kidneys, under the microscope, show marked thickening of the walls of the blood vessels and of the capsules of Bowman. There is distinct round cell infiltration about the latter and, to a less extent, about the thickened vessel walls. The tubules in the pyramids are normal, the epithelium being intact and clean, with well marked and clearly stained nuclei. The epithelium of the cortex is very uniformly swollen, granular and in some places desquamated. I should say that the kidney had been the seat of a long standing interstitial inflammation, and had had a recent parenchymation implaced upon it from some infection. There was no evidence of fatty degeneration in any part of the section, even on staining with osmic acid. No micro-organisms could be demonstrated in the tissues by means of carbol fuchsine staining.

" 'The liver likewise showed a slight increase of interlobular connective tissue, but not to the same degree as could be seen in the kidneys. The epithelium was slightly swollen and granular, but the nuclei were still fairly colorable with ordinary stains. No evidence of fatty degeneration even with osmic acid could be seen.

" 'A culture was obtained from the patient's blood during life, upon agar. Thus far we have been unable to get secondary growths after numerous attempts upon other media, although in several tubes ordinary mold fungi have been planted along with the inoculation. The growth at 60 degrees temperature appeared as a small, colorless, round, transparent colony, with a slightly opaque border, the same apearance remaining in either degrees or growth in the same tube. It has never lost this thin, same like appearance after changing the temperature to that of the room, and has in no wise appeared like the drop of sealing wax described by Sanarelli as characteristic of his bacillus cultures. A very faint fluorescence appears in the media close to the colony. Under the microscope the individuals are seen as small rods, from 1.5 to 2 m. m. long and from .25 to .5 m. m. wide, stained with the common aniline solutions and taking the stains more deeply at the ends than in the middle. No motion can be noted. The difficulty experienced in obtaining secondary growths of these bacilli has thus far prevented a detailed study of their characteristics, which is to be hoped may later be successfully done.

"'Very truly,

"'Allen J. Smith.'"

*Now contrast these notes from Ocean Springs case:

Necropsy eight hours after death, body of a fairly nourished female, rigor mortis well marked; conjunctiva, pale; eyes, golden yellow; body, jaundiced; hypostatic congestions observed around the neck, back and shoulders.

Left lung adherent to chest walls, old adhesions; tubercles at base.

Right lung, adherent, cavities.

The pericardium was filled with normal fluid.

Heart, normal in size, muscular tissue indicated to the eye fatty degeneration.

Liver, pale boxwood color, normal in size, quite friable, easily torn; showed fatty degeneration.

Gall bladder filled with bile.

Stomach distended, contained about six ounces of black fluid. Extravasation of blood into the tissue of membrane, mucous membrane congested, vessels congested, serious coat of a yellowish tint.

Pancreas normal. The omental and peritoneal vessels congested.

The intestines, mucous membrane and vessels same condition as met with in the stomach.

Spleen normal in size and consistence.

Left kidney somewhat enlarged, somewhat softened. Cortical substance, yellowish tint; showed signs of fatty degeneration.

Right kidney normal in size, otherwise same as left.

Bladder normal.

The black vomit examined microscopically showed a considerable number of blood corpuscles and epithelium and disintegrated food. Also the streptococci (Sternberg), which I have been able to cultivate on agar.

Microscopical examination of liver shows that the hepatic cells are almost completely hidden by fat globules.

Kidneys examined microscopically show the presence of fat globules in cells covering epithelial layer of glomeruli and also in the uriniferous tubules. Some tubules are denuded of their epithelial lining.

*And these from the Gelpi case:

Pathological History.—Notes from the autopsy. General appearance: Whole body of distinct lemon-yellow color: ecchymoses generally, more marked over the posterior half of the body; conjunctivae also distinctly yellow; bloody froth oozing from the mouth and nose; liver yellowish in tint and showing distinct signs of fatty degeneration. Kidneys: both very much congested and enlarged, distinct degeneration, though slighter than in the liver; both quite hemorrhagic on section. Spleen: not enlarged apparently, but showing signs of degeneration. Stomach: mucous lining much congested and swollen, and cavity containing bloody fluid thicker than that coming from the mouth.

Report of Dr. O. L. Pothier, Pathologist and Bacteriologist, Charity Hospital.—I beg to report as follows on microscopical examination of organs from autopsy on body of Mr. Gelpi. Liver: found liver cells undergoing fatty degeneration; in areas of section this degeneration was very marked, leaving surrounding tissue less diseased. Many of the blood vessels were still filled with blood. Kidneys: almost all of the renal epithelium was degenerated, though this was more marked in some areas; the epithelial cells were markedly granular, staining badly everywhere, the nuclei in some barely visible the whole cell appearing occasionally as a mass of granular matter; some of the tubules were occluded with this material. Spleen: marked increase in lymphoid element; areas of fatty degeneration also found, but this degeneration seemed more localized to different areas, while surrounding tissue in immediate neighborhood appeared healthy. Stomach: except for the extravasation of blood in the coats of the stomach, especially in part of mucous membrane near glands and between them, and for the brownish discoloration at surface of mucous membrane, there was no inflammatory and degenerative change.

The last two Sternberg and Schmidt would diagnose as yellow fever.

The first no one could determine to be of that nature. Dr. Allen J. Smith so expressed himself.

---

*New Orleans Medical and Surgical Journal, December, 1897.

Wade may not have had Weil's disease, but certainly his case did not exhibit the pathological changes of yellow fever.

Note especially the absence of steatosis in the Wade case.

Now I shall pass over the suggestion that dengue fever in the light of the experience of 1897 is properly a quarantinable disease, and submit the proposition that if we read the teachings of this epidemic clearly and fairly we will be compelled to conclude that either yellow fever was not present during this epidemic, or if it was present its behavior justifies the position that it is not for us a quarantinable disease; for evidently the infection does not propagate and spread at this day in this section.

Independent of any such consideration, with any reference to the epidemic just past, I desire to say that I believe the tendency of modern medicine is rapidly towards the position which will relegate quarantine certainly between cities of this country on account of yellow fever to the past. I believe the day is coming when the disease will be isolated in a community through disinfection practiced along scientific lines, and the infection die and disappear without spreading. And when a knowledge of the disease can take away its element of contagiousness, as has already been done noticeably in the hospitals in New Orleans during the last season, the popular mind will cease to fear it, the wheels of commerce will not be stopped, the marts of trade will know no interruption, and the mass of the people who work for their daily bread will not find themselves face to face with the alternative of starvation or a dreaded scourge.

Dr. R. T. Morris: In reply to Dr. Lee's very able discussion I will state that he, like others who had similar views in regard to the late epidemic, begs the question, evades the issue. He contends that there was no yellow fever, but he does not show how dengue fever can possess the symptoms that were present.

A rational diagnosis of any disease is based upon history and symptomatology, and it is a bold man, indeed, who will ignore the experience and observation of medical authorities.

I have attempted persuasion, cajoling, and I have even demanded that they show me one authority to sustain them in the assertion that dengue can possess the symptoms that were observed in the so-called anomalous cases. Holliday does not, neither does Mahe, Holliday, Foster, Matis or McLaughlin; and it is not disputed that albuminuria, divergent pulse and temperature, and jaundice were observed in the suspicious cases, nevertheless many contend that it was only aggravated dengue.

Dr. Lee doubts that the suspicious cases were yellow fever, because those in contact with them or under the same roof did not develop a suspicious fever.

In Houston several cases could be traced to foci of infection. A well known physician in Houston had under his charge several suspicious cases in one family. They were exceedingly anomalous and albumen was in abundance. Later the physician developed a suspicious case, peculiar facies, hemorrhages from the bowel and albuminous urine. Again a young man nurses a friend, whose case is anomalous, and later the amateur nurse develops an anomalous case.

In Turo Infirmary (N. O.), fully 100 non-immunes were with the yellow fever cases and no case developed in the hospital; fully the same number of non-immunes were exposed to the disease in the Isolation Hospital (N. O.) and none contracted the disease, although the ambulance driver, a non-immune, frequently came in contact with black vomit.

Dr. Lee objects to my mortuary table published in the paper I read at Beaumont. This table was copied from the records in the city physician's office and its accuracy can not be reasonably doubted. As to the fatal case which was of four days' duration, with jaundice, divergent pulse and temperature, albuminuria and black vomit; I will state that he was in a first class condition previous to the sickness, I examined him carefully not less than three weeks before and I can assert that his organs were normal and that there was no albumen in the urine and no indication of kidney trouble.

While I have a high opinion of Dr. Lee's discussion, I believe he has failed to sustain his position. In the light of past epidemics of yellow fever and in view of the literature concerning both dengue and yellow fever, there is only one interpretation of the anomalous cases and that is that they were yellow fever.

Dr. H. A. West: To properly reply to Dr. Lee would involve the second reading of my paper, but as you are probably as much exhausted from the hearing as I am from the reading I will make no such attempt. There are, however, a few points to which I beg to call your attention. I am very happy to know that Dr. Lee thinks I have been fair and conservative, though he damns me with faint praise by regretting the paper was not more comprehensive. To show that I have attempted at least to be fair, I will state that I submitted my MS. to the doctor in advance that he might read, mark, inwardly digest, and possibly learn something about the subject. I am sorry that I can not return the compliment as to fairness upon the part of Dr. Lee, as I will proceed to show: 1st, Dr. Lee accuses me of moving in a circle and returning to the position of Dr. Guiteras when I state that granting the reliability of the testimony presented in the tabular report that in dengue simple parenchymatous changes may occur in the kidneys and be manifested by an evanescent and mild albuminuria while in yellow fever a series of cases will afford incontestible evidence of a severe nephritis, viz: scanty urine, of high color and specific gravity, intense and persistent albuminuria, hematuria, casts, decided tendency to suppression, and the accompanying uremia. If this is reasoning in a circle, or returning to the position of Dr. Guiteras, I know nothing of logic or the force of the English language, and it is unfair to make such an accusation. Moreover, I defy Dr. Lee or any one else to show a single authority for the statement that evidence of a serious kidney involvement belongs to the symptomatology of dengue. 2d. Dr. Lee says: "Passing on to Dr. West's hypotheses I shall not discuss the suggestion that the epidemic was yellow fever only. No one believes so." And yet we find Dr. Lee, in a paper published in the New Orleans Medical and Surgical Journal, discusses the subject under the title "Dengue or Yellow Fever?" What does he mean by this interrogative title? Either that in his own opinion the epidemic must be one thing or the other, and that there was no ground for those to stand upon who contend that the two diseases might exist concurrently, or he imputed this opinion to others. I submit that it is unfair now for him to say "no one believes so." 3d. Dr. Lee presents the notes of the autopsy in the Wade case to prove that it was not yellow fever, and states that Dr. Allen J. Smith so expressed himself, whereas to the contrary, Dr. Gammon, in reply to my question on this floor, informs us that Smith's opinion was that Wade died of yellow fever, and moreover that investigation subsequent to the autopsy demonstrated the presence of an organism corresponding in all essential particulars with Sanarilli's icteroid bacillus. Is it fair to quote Dr. Smith as having an opinion when Dr. Lee had every opportunity to know he had a different one? 4th. Dr. Lee gives the pathological changes in two of the Ocean Springs cases, leading us to infer that such changes must necessarily and universally be present in every person dying of yellow fever, which claim I do not think can be fairly made. The history of the Wade case gives the complete symptomatology of yellow fever, Dr. McLaughlin lectured on it as a typical case, Dr. J. E. Burke thought it was that disease until after the autopsy, and I submit that it is not fair to negative that diagnosis simply upon the absence of hepatic steatosis.

## Figures and Tables

**Figure f1:**